# Damages for Vaccination

**Published:** 1873-11

**Authors:** 


Damages for Vaccination. — A case recently came up for adjudication in the courts which has something of a medical interest attached to it.

In July, 1871, when the small-pox prevailed to an unusual extent in this city, and there was fear of it becoming an epidemic, the Board of Health detailed certain surgeons to vaccinate throughout the city all who had not been vaccinated. Dr. Brown, one of such surgeons, vaccinated Alice E. Hallowell, then four years old. It is claimed that through such vaccination she was inoculated with scrofula, and a suit was accordingly brought by the father of the girl against the city to recover $10,000 damages. The testimony for the father showed that he had forbidden his wife to let the child be vaccinated by any public physician, but that Dr. Brown disregarded this injunction and insisted on the vaccination. Several physicians also testified that scrofula, syphilis, and various other diseases might be imparted through the use of improper vaccine matter. At the close of the testimony for the plaintiff, a motion was made by the defense to dismiss the complaint, and the same was argued at considerable length. It was urged that the Doctor exceeded his authority, but the main point insisted upon was, that the city was not liable in the case, in as much as the Board of Health under the present laws was distinct from the city government. Judge Van Brunt took this view of the case, and dismissed the complaint.

